# Isolation and Antimicrobial Activities of Phytochemicals from *Parinari curatellifolia* (Chrysobalanaceae)

**DOI:** 10.1155/2021/8842629

**Published:** 2021-03-04

**Authors:** Phillip Mawire, Winnie Mozirandi, Matthias Heydenreich, Godloves Fru Chi, Stanley Mukanganyama

**Affiliations:** ^1^Department of Biochemistry, University of Zimbabwe, Mt. Pleasant, Harare, Zimbabwe; ^2^Universität Potsdam, OT Golm, Institut für Chemie, Haus 25, D/0.19 (Labor E/0.06-0.08), Karl-Liebknecht-Str. 24-25, D-14476 Potsdam, Germany; ^3^Department of Organic Chemistry, University of Yaoundé I, P.O. Box 812, Yaoundé, Cameroon

## Abstract

The widespread use of antimicrobial agents to treat infectious diseases has led to the emergence of antibiotic resistant pathogens. Plants have played a central role in combating many ailments in humans, and *Parinari curatellifolia* has been used for medicinal purposes. Seven extracts from *P. curatellifolia* leaves were prepared using serial exhaustive extraction of nonpolar to polar solvents. The microbroth dilution method was used to evaluate antimicrobial bioactivities of extracts. Five of the extracts were significantly active against at least one test microbe. *Mycobacterium smegmatis* was the most susceptible to most extracts. The methanol and ethanol extracts were the most active against *M. smegmatis* with an MIC of 25 *µ*g/mL. The hexane extract was the most active against *Candida krusei* with an MIC of 25 *µ*g/mL. None of the extracts significantly inhibited growth of *Klebsiella pneumoniae* and *Staphylococcus aureus*. Active extracts were selected for fractionation and isolation of pure compounds using gradient elution column chromatography. TLC analyses was carried out for pooling fractions of similar profiles. A total of 43 pools were obtained from 428 fractions. Pools 7 and 10 were selected for further isolation of single compounds. Four compounds, Pc4963r, Pc4962w, Pc6978p, and Pc6978o, were isolated. Evaluation of antimicrobial activities of Pc4963r, Pc4962w, and Pc6978p showed that the compounds were most active against *C. krusei* with MFC values ranging from 50 to 100 *µ*g/mL. Only Pc6978p was shown to be pure. Using spectroscopic analyses, the structure of Pc6978p was determined to be *β*-sitosterol. The antifungal effects of *β*-sitosterol were evaluated against *C. krusei in vitro* and on fabrics. Results showed that *β*-sitosterol reduced the growth of *C. krusei* attached to Mendy fabric by 83%. Therefore, *P. curatellifolia* can be a source of lead compounds for prospective development of novel antimicrobial agents. Further work needs to be done to improve the antifungal activity of the isolated compound using quantitative structure-activity relationships.

## 1. Introduction

Infectious diseases remain the major cause of morbidity and mortality especially in the developing countries [1]. Resistance to common antimicrobial agents has led to high frequency of treatment failures and increase in treatment cost [2]. These incidences have resulted in prolonged hospital stay, leading to an increase of nosocomial infections [3, 4]. The spread of antimicrobial resistance shows that even newly designed antimicrobial agents will have brief life expectancy of efficiency [5, 6].

Microbial resistance is reported to be acquired from different environmental sources including waste water, hospital surfaces, and contaminated textiles [7–9]. Clothes are often contaminated with certain pathogens when being worn or even after being washed [10]. Some bacteria tightly stick to the textile and tend to form biofilm which is difficult to remove [11], where resistance can be developed [7]. Contaminated textiles can cause health problems in the hospital environment [11]. The role of contaminated textiles, environmental surfaces, and medical devices in the transmission of health-associated pathogens and the development of microbial resistance has been well reported [12, 13]. There is a need for efficient therapy for bacterial diseases [14] and to search for alternative compounds that can be used as surface and textile decontaminates [15].

Medicinal plants have been identified as important sources of natural antimicrobial compounds [16] and biocides [15]. Therapeutic value of these plants lies in some bioactive substances that produce a distinct physiological activity on the human body [17]. The most significant of these active phytochemicals are alkaloids, tannin, flavonoid, sterols, and phenolic compounds [18]. These compounds can be an alternative in treating microbial infections [6], as well as in formulation of alternative disinfectants [19].

*P. curatellifolia* is one of the most important medicinal plants for the people of Zimbabwe [20]. This plant has various documented uses in ethnomedicine including treatment of wound infections, cancer, pneumonia, fever, bacterial infections, and inflammation [21]. A number of studies have shown that extracts from *P. curatellifolia* have a number of pharmacological properties ranging from antibacterial activity to anticancer activity [20]. The pharmacological properties of *P*. *curatellifolia* are linked to the presence of pharmacologically active phytochemicals including saponins, flavonoid, tannins steroid, alkaloid, cardiac glycosides, carbohydrate, balsams, and terpenes [22]. Although there are a number of studies on the pharmacological properties of *P*. *curatellifolia* extracts, there has been a paucity of documented data on antimicrobial properties of pure compounds isolated from *P. curatellifolia*.

To date, several methods have been utilized when preparing samples to determine the antimicrobial action of plants [1]. These include preparation of crude extract using a variety of solvents, purification of active compounds from extracts, subsequent extraction with increasing solvent polarity, and distillation [16]. However, recent studies have shown enhanced activity of plant samples following fractionation of the extract and further purification of compounds [1, 22]. The effects of *β*-sitosterol contaminated textiles were also assessed in an activity-guided procedure. *β*-Sitosterol is a natural phytosterol found in the cells and membranes of all oil producing plants [23]. *β*-Sitosterol has been shown to be a safe, nontoxic, effective nutritional supplement and has potential health benefits in many diverse clinical applications [24]. These include anti-inflammatory activity, chemopreventive effects, angiogenic effects, and antidiabetic effects [25].

The aim of this study was to isolate active compounds from *P. curatellifolia* and determine the antimicrobial activities of the isolated compounds on some common microbes implicated in nosocomial infection [26, 27] as well as on contaminated textiles. Isolation of these compounds may give a chance to evaluate the antimicrobial effects of the individual compounds as well as their synergistic effects with commercial antibiotics that are already in use. Isolated compounds from this study can then be used as templates for the production of new microbial agents or as supplements to the existing antimicrobial agents.

## 2. Materials and Methods

### 2.1. Plant Material

Fresh leaves of *P. curatellifolia* were collected between June and July of 2017 from Norton (latitude -17.88; longitude 30.70; situated at elevation 1364 meters above sea level) in Mashonaland west Province of Zimbabwe. The plant samples were authenticated at the National Herbarium (Harare, Zimbabwe) where a voucher specimen was made and deposited under the reference number C6E7. Permission to use the plant samples was granted by the Faculty of Higher Degrees Committee, Harare, Zimbabwe (HD/71/16).

### 2.2. Growth of Microbial Cells

Four microbial isolates drawn from the list of common and emerging nosocomial pathogens were used in the study [25, 26]. *M. smegmatis* 155 mc^2^ laboratory strain was obtained from the Department of Clinical Laboratory Sciences, University of Cape Town. A clinical strain of *C. krusei* and *K. pneumoniae* (ATCC 700603) was obtained from the Department of Medical Microbiology, University of Zimbabwe*. Staphylococcus aureus* (ATCC 9144) was obtained from the Department of Biological Science at the University of Botswana (Gaborone, Botswana).

### 2.3. Culture Media and Chemicals

All chemicals, reagents, and culture media used were purchased from Sigma-Aldrich Chemical Company (Taufkirchen, Germany). Organic solvents were distilled prior to use. *K. pneumoniae* and *S. aureus* were grown and maintained in Luria broth and Luria agar. *M. smegmatis* cells were grown and maintained in Middlebrook 7H9 broth supplemented with 1 g/L casein acid hydrolysate and Middlebrook 7H10 agar supplemented with 1 g/L casein acid hydrolysate. *C. krusei* cells were grown and maintained in Sabouraud dextrose broth and agar.

### 2.4. Preparation of Plant Extracts

The leaves were washed under running distilled water to remove any dust and grit and then air-dried at room temperature. Leaf powder was prepared manually by hand pounding dry leaves using a wooden pestle and mortar. Phytochemicals from the leaf material were extracted by maceration using the DCM-MeOH extraction and the serial exhaustive extraction methods successively [28].

### 2.5. Extraction of Phytochemicals with Dichloromethane/Methanol

Phytochemicals from 1500 g of powdered *P. curatellifolia* leaf material was extracted in 4500 mL of a solution of 50%:50% dichloromethane (DCM) and methanol, respectively, for the total extraction. The mixture was left for extraction for 72 hours with constant shaking. The extraction mixture was filtered through cotton wool and then through Whatman No. 1 paper. The leaf debris (residue) was air-dried and stored at room temperature for further extraction. The filtrate was concentrated using a rotary evaporator RII (BUCHI, Labortechnik AG, Switzerland) at 50°C and low pressure. The extracts were dried to a constant mass under a fan in a fume hood cabinet. Dried extracts were stored in sterile screw cap tubes at 4°C until use.

### 2.6. Serial Exhaustive Extraction

Extraction was done sequentially using different solvents on the leaf debris (residue) from the dichloromethane-methanol mixture (50% *v*/*v*) (DCM-MeOH). The serial exhaustive extraction was done in the following sequence of solvents: hexane, DCM, acetone, ethyl acetate, ethanol, methanol, and water. At each extraction stage, the leaf debris generated after filtration was left to dry in a sterile fume hood; after drying, the debris was used in the subsequent extraction stage. The filtrate was concentrated using a rotary evaporator RII (BUCHI, Labortechnik AG, Switzerland) at 50°C and low pressure. The extracts were dried to a constant mass under a fan in a fume hood cabinet. Dried extracts were stored in sterile screw cap tubes at 4°C until use. The weight of dry extract obtained was recorded and percentage yield was determined.

(1) $$\begin{array}{c} \text{extraction yield}\left(\%\right)=\frac{\text{weight of dried plant extract }\left(\text{g}\right)}{\text{weight of dried plant material}\left(\text{g}\right)}\times 100\%. \end{array}$$

Crude extracts were collected in vials for further analysis.

### 2.7. In Vitro Antimicrobial Activity

#### 2.7.1. Preparation and Standardization of Inocula

The inoculum of each microbe was prepared by reviving cell from glycerol stocks. Cells for the assay were standardized according to the 0.5 McFarland solution, which is equivalent to 1.5 × 10^8^ CFU/mL.

#### 2.7.2. INT Colorimetric Assay for Minimum Inhibitory Concentrations (MIC)

**Figure 1:**
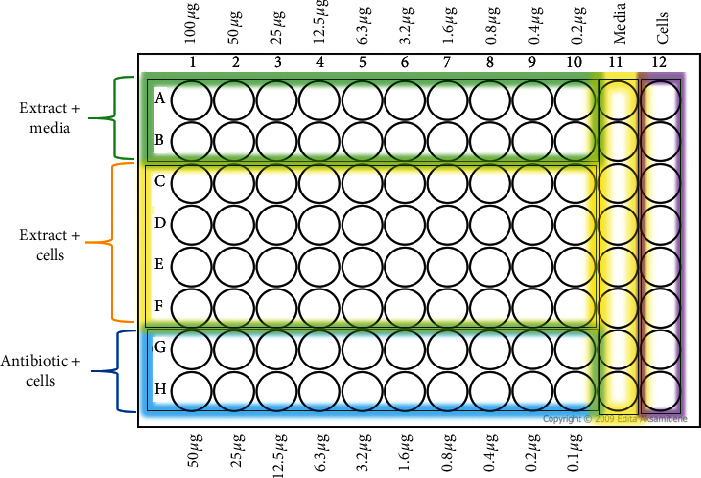
Plate setup for 2-(4-iodophenyl)-3-(4-nitrophenyl)-5-phenyl-2H-tetrazolium chloride (INT) colorimetric assay for minimum inhibitory concentrations (MIC). The “cells” column is the positive control for microbial growth (cells were inoculated in untreated broth media). The “media” column is the negative control for cell growth (wells contained uninoculated broth media). The “antibiotic + cells” rows are the antibiotic/antifungal positive control (cells were inoculated in wells with decreasing standard antimicrobial/antifungal concentration). The “extract + cells” rows are the test wells (cells were inoculated in wells with decreasing extracts concentrations).

The inhibitory potential of microbial growth by both the crude extracts and the pure compound was determined by broth microdilution method in 96-well microtiter plates [29]. The MIC assay was conducted using 2-(4-iodophenyl)-3-(4-nitrophenyl)-5-phenyl-2H-tetrazolium chloride (INT) colorimetric assay according to Eloff [30] with slight modifications. The assay for minimum inhibitory concentration was conducted according to the plate layout shown in Figure 1. Working concentration of test samples was prepared by dissolving 0.004 g of the extracts of isolated compound in 1 ml dimethylsulfoxide (DMSO) to make a concentration of 4 mg/ml. Then, 0.5 ml was taken from the working concentration and made up to 10 ml by adding sterile broth media to make a test concentration of 200 *µ*g/ml. DMSO was used to prepare the samples was 2.5%. A serial twofold dilution was performed to obtain extract concentrations ranging from 0.4 to 200 *µ*g/mL using sterile broth. Liquid cultures of each test microbe were grown in broth media. These were diluted in fresh broth, and 100 *µ*L was applied to the wells of a 96-well plate. In each case, approximately 1.5 × 10^8^ CFU/mL of exponentially growing cells were inoculated for each strain. The extracts (100 *µ*L) were added to these wells in decreasing concentrations and mixed by pipetting. Each well was further diluted with 100 *µ*L microbial inoculum. The final extract concentration in the 96-well plate ranged from 0.2 to 100 *µ*g/mL. Cells in wells with unexposed broth were used as the positive control for microbial growth, while wells with plain broth were used as negative cell growth (Figure 1). Cell density of the plate was measured at 590 nm using a microplate reader (Tecan GENios Pro, Grödig, Austria) prior to incubation. The plate was incubated at 37°C for 18 hrs. Cell density was measured after incubation. Growth of cells was determined by calculating the difference of the preincubation value from the postincubation value. Data are presented as percentage inhibition of inoculum. Percentage inhibition was obtained using the following equation:

(2) $$\begin{array}{c} \text{percentage inhibition}\left(\%\right)=\frac{\text{positive control value}-\text{sample value}}{\text{positive control value}}\times 100. \end{array}$$

Following incubation, 50 *µ*L of 0.2 mg/mL INT was added in each well. The plates were incubated for an additional 30 min at 37°C. Viable microbes reduced the yellow dye of INT to a pink color. MIC values were defined as the sample concentration that exhibited complete inhibition of bacterial growth, that is, the sample concentration that prevented the color change of INT [30].

#### 2.7.3. Minimum Bactericidal/Fungicidal Concentration (MBC/MFC) Determination

The MBC/MFC was determined after MIC assays [31]. The MBC/MFC of the sample was determined by subculturing 50 *µ*L of the suspensions from the wells that did not show any growth after incubation during MIC assays on an agar plate. The inoculated agar plates were incubated at 37°C for 24 hours before observation. The MBC/MFC was defined as the lowest concentration of a sample that completely inhibited the visible microbial growth on the agar plates. Each assay was performed in triplicate.

### 2.8. Chromatographic Separation of Extracts

Active extracts against at least one of the test organisms were selected and combined. Combined extracts were fractionated using the gradient elution column chromatography. In this study, the methanol extract, DCM:MeOH extract, ethanol extract, and hexane extract were selected for chromatographic separation.

#### 2.8.1. Gradient Elution Column Chromatography

Selected extracts were combined and adsorbed on equal mass of silica (70 g of silica, 60–120 mesh) to make a loading sample. The sample was packed on a silica gel column, and the gradient elution method was used to run the column. The column was first eluted with n-hexane as the mobile phase, and the polarity of the mobile phase was increased by gradually adding 2.5% of ethyl acetate increments. After reaching 100% ethyl acetate, the polarity was further increased by gradually adding 2.5% methanol. Volumes of 250 mL fractions of the eluate were collected during the process. A total of 428 fractions were obtained from the column chromatography, and each fraction was concentrated using a rotary evaporator RII (BUCHI, Labortechnik AG, Switzerland). The fractions were dried to a constant mass under a fan in a fume hood cabinet. All fractions were stored in sterile screw top tubes at room temperature.

#### 2.8.2. TLC Profiling of Chromatographic Fractions

Concentrated fractions were subjected to thin layer chromatography (on aluminium plate coated with Mercy, silica gel 60 F254). TLC plates were developed for each fraction in a saturated tank with a mobile phase of slightly higher polarity than used in column chromatography to elute the fraction. For example, fractions eluted in 100% hexane were developed in 97.5:2.5 (HEX:EA) solution. Developed chromatograms were located by observation under short and long ultraviolet light (short UV and long UV at 254 nm and 360 nm, respectively), then sprayed with 10% *w/v* sulphuric acid, and dried at 100°C. Fractions showing similar profile were pooled into the same pool. Pooled fractions were dried in glass vials and their yields determined. Pooled fractions were then kept at room temperature for further analysis.

### 2.9. Purification of Compounds Using Preparative TLC

Fractions showing the best TLC separation as well as sufficient yield were selected for further purification of single compounds using the preparative TLC. In this case, pools 7 and 10 were selected for the isolation of single compounds. Preparative TLC was made by absorbing the extracts on 2 mm layer of silica Type G with 13% calcium sulphate on 20 × 20 cm glass plate. The plate was activated by heating in oven at 60°C for 30 minutes prior to use. The selected pools were combined, dissolved in chloroform, and then loaded on the activated TLC plate. The plate was allowed to develop in a chloroform-saturated tank. Once developed, the separated chromatograms were observed under UV light at 254 nm using a UV lamp in a UV-viewing cabinet. At least four chromatograms of different colors were separated. Silica at the position showing a single isolated chromatogram on the plate was scraped off using a metal spatula blade. The chromatogram content on the scraped silica was eluted using chloroform through a Whatman No. 1 filter paper into four different glass vials. The eluent was then evaporated at room temperature and then stored as solids in the vials. The crystallized solids in the vials were further analyzed for purity using TLC with chloroform as the mobile phase. The solids that gave a single TLC chromatogram confirmed the purity of the isolated compound. Using TLC, only one compound out of four was confirmed to be a pure compound. Antimicrobial evaluation of the pure compound was determined against the test microbes. NMR analyses and determination of the mass of the isolated compound were carried out.

### 2.10. NMR Analyses and Determination of the Mass of the Isolated Compound

**Figure 2:**
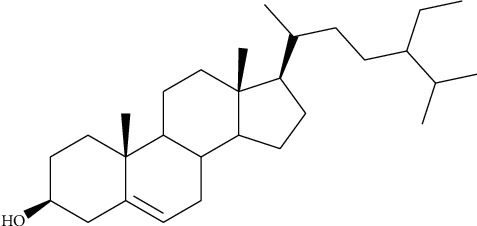
Structure of *β*-sitosterol.

The ^1^H NMR spectra were recorded at 400 MHz and ^13^C NMR spectra at 100 MHz. The chemical shifts for ^1^H NMR and ^13^C NMR were referenced to TMS via residual solvent signals (^1^H, CDCl_3_ at 7. 26 ppm; ^13^C, CDCl3 at 77.36 ppm; ^1^H, DMSO*-d*_6_ at 2.45 ppm; ^13^C, DMSO*-d*_6_ at 39.43 ppm; ^1^H, CD_3_OD at 3.31 ppm; ^13^C, CD_3_OD at 49.0 ppm). The 2D NMR experiments were run using standard pulse sequences. Molecular formulae were determined by electrospray ionization with a 7-T hybrid ion trap and a TOF detector running in positive or negative mode. *β*-Sitosterol (Figure 2) was identified as one of the pure compounds, and its effects on the test organisms were evaluated.

### 2.11. Biocidal Activity Assays of the Most Active Compounds on Mendy Fabric

Biocidal evaluation of the purified compounds was done on the Mendy fabric, which is the material used to make uniforms for nurses in Zimbabwe. The biocidal evaluation was done using a simple and rapid method for optical visualization and quantification of bacteria on fabrics using the INT dye, by Stiefel et al. [10] with some modifications. Absorbance measurements for the biocidal activity assay were done using a Shimadzu UV/VIS UV-1601 spectrophotometer (Shimadzu, Kyoto, Japan). Standard curves to correlate the staining intensity with the numbers of microbial cells were first generated. The standard curves were used to determine the number of bacterial and fungal cells left after the microbial contaminated fabric was treated with the isolated compound.

To generate the standard curves, 100 *µ*L microbial culture of different concentrations (OD_600_: 0, 0.125, 0.25, 0.375, and 0.5) was directly applied to the fabric samples in 6-well plates. Samples were incubated for an hour at 37°C. After incubation, 1 mL of media and 0.2 mg/ml 2-(4-iodophenyl)-3-(4-nitrophenyl)-5-phenyl-2H-tetrazolium chloride (INT) was added to each fabric (staining with tetrazolium salt). The sample was incubated for 30 minutes at 37°C and with shaking at 40 rpm. After incubation, the liquid was removed, and 1 mL dimethyl sulfoxide (DMSO) was added to each well. The dye was removed by pipetting the mixture several times onto the fabric until a homogenous color was obtained. Absorption of the eluate was measured at 490 nm using DMSO as a blank. As a background control the different fabrics were treated in the same way using media without bacteria.

To correlate OD_600_ values of the applied culture with cell numbers, colony forming units (CFU) were determined at the same time point as the staining of bacterial culture. The same bacterial solutions as added to fabrics were also incubated for 1 hour at 37°C and 80 rpm. After 1 hour, the bacterial culture was used to determine cell numbers by plating a 1:10 dilution series on agar. The CFU of appropriate dilutions were counted after 1 day, and the exact cell numbers corresponding to the applied culture were determined. The bacterial culture with an OD_600_ value of 0.5 corresponded to about 3.15 × 10^10^ bacteria per square centimetre of fabric for *M. smegmatis* cells and 5.6 × 10^8^ fungal cells per square centimetre for *C. krusei*. All the standard curves were generated using GraphPad Prism (Version 5.03, GraphPad Software Inc., San Diego, California, USA).

To evaluate the biocidal activities of the compounds on the Mendy fabric, 100 *µ*L of the microbial culture with an OD_600_ of 0.5 in broth media was directly applied to the fabric samples in 6-well plates and incubated for 1 hour at 37°C. After incubation, 100 *µ*L of (200 *µ*g/mL) test compound was directly applied to the fabric samples in the 6-well plates and incubated for 30 minutes at 37°C. Concentrations of 100 *µ*g/mL and 50 *µ*g/mL of chlorhexidine digluconate were applied to pieces of the same fabric in different wells as the biocide positive control. After incubation, 1 mL growth medium containing 0.2 mg/mL INT was added to the fabric without removing unbound bacteria and incubated for 30 minutes at 37°C and 40 rpm. The dye was eluted from the fabric using 1 mL DMSO and pooled with the supernatant, in order not to lose the unbound bacteria. Staining intensity was quantified by measuring absorbance at 490 nm using a Stat Fax 2100 microplate reader (Awareness Technologies Inc., Westport, USA). For background control, media without bacteria were used during incubation. The number of viable cells that remained on the fabric was quantified by comparing the measured signal intensity to the standard curve.

### 2.12. Statistical Analysis

Statistically significant differences between the mean of the controls and the tests were analyzed using one-way ANOVA with Dunnett's multiple comparison posttest. Enzyme kinetics were analyzed with linear regression using GraphPad Prism (Version 5.03, GraphPad Software Inc., San Diego, California, USA).

## 3. Results

### 3.1. Extraction Yields of the Plant Extracts

The highest yield, 3.53%, was obtained from extraction of *P. curatellifolia* leaves with the dichloromethane (DCM) and methanol, while the least yield, 0.23%, was from the hexane extraction (Table 1).

### 3.2. In Vitro Antimicrobial Activity of the Extracts

**Figure 3:**
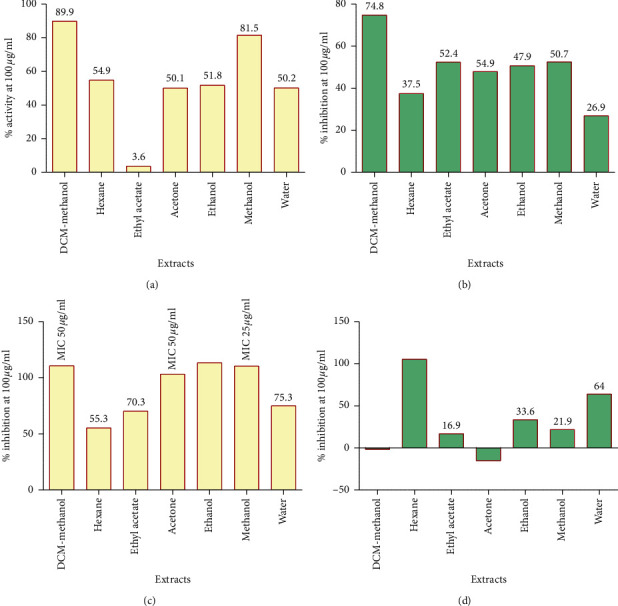
Percentage inhibition of the crude *P. curatellifolia* extracts at 100 *µ*g/mL on the growth of *S. aureus* (a), *K. pneumoniae* (b), *M. smegmatis* (c), and *C. krusei* cells (d).

Evaluation of the antimicrobial activities of the extracts against the four test organisms was done using the INT colorimetric assay for minimum inhibitory concentrations. The study has shown that all the extracts used in the study exhibited varying degrees of antimicrobial activity against all the microorganisms tested (Table 2). The DCM-methanol extract had the highest activity against *S. aureus*, reducing cell growth by 90% after incubation. However, the ethyl acetate extract had the least activity of only 3.6% against *S. aureus* (Figure 3(a)). There was no complete inhibition of growth for *S. aureus* and *K. pneumoniae* by any of the crude extracts even at the highest concentration, 100 *µ*g/mL, that was tested (Figures 3(a) and 3(b)). However, the DCM-methanol extract had the highest activity, 75%, against the *K. pneumoniae,* while the water extract had the lowest activity, only 26.9%, after incubation (Figure 3(b)). *M. smegmatis* was more susceptible to all the *P. curatellifolia* extracts compared to other test microbes (Table 2). The ethanol and methanol extracts had the highest activity of MIC, 25 *µ*g/mL, against the *M. smegmatis,* followed by the DCM-methanol and the acetone extract which had MIC of 50 *µ*g/mL each on the same cells (Figure 3(c)). The DCM-MeOH had an MBC value of 50 *µ*g/mL while the methanol extract had an MBC value of 100 *µ*g/mL against *M. smegmatis* (Table 2). Only the hexane extract and water extract were able to inhibit the growth of *C. krusei* cells by more than 50% at a concentration of 100 *µ*g/mL after incubation (Figure 3(d)). The DCM-methanol and the acetone extract completely failed to inhibit growth of *C. krusei* (Figure 3(d)). The hexane extract was the most effective in inhibiting growth of *C. krusei* with an MIC value of 25 *µ*g/mL after incubation (Figure 3(d)), but no extracts exhibited MFC against *C. krusei* (Table 2).

### 3.3. Chromatographic Separation of Extracts

**Figure 4:**
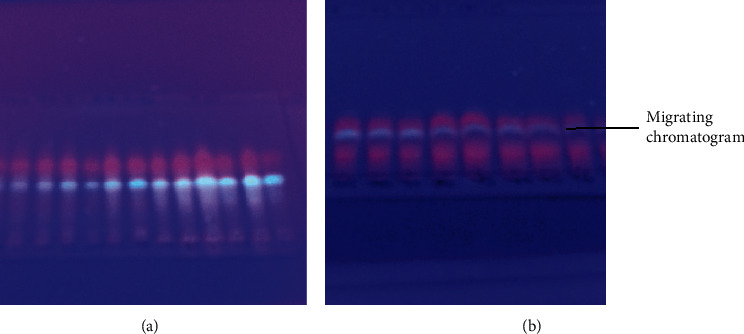
TCL chromatogram showing similar fractions constituting pool 7 (a) and pool 10 (b).

**Figure 5:**
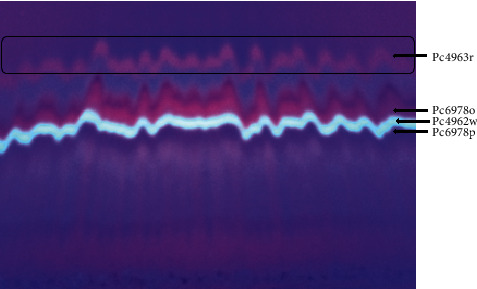
Preparative TLC chromatogram. The chromatogram shows that at least four chromatograms (Pc4963r, Pc4962w, Pc6978p, and Pc6978o) migrated to different positions as visualized under UV.

The extracts that had potent antimicrobial activity are as follows: DCM-methanol extract, hexane extract, ethanol extract, and ethanol extract by inhibiting growth of at least one test organism (Table 2) were selected for chromatographic separation. Selected extracts were combined and fractionated using gradient elution column chromatography. A total of 43 distinct pools were obtained from 428 fractions. The pools were numbered from 1 to 43. Pools 7 and 10 (Figure 4) were observed to exhibit better separation of chromatogram on TLC plate. Pools 7 and 10 were selected for further separation of single compounds using preparative TLC. At least four chromatograms with different colors, red, white, dark red, and purple, were obtained. The chromatograms were assigned code names Pc4963r, Pc4962w, Pc6978o, and Pc6978p, respectively, for identification (Figure 5).

**Figure 6:**
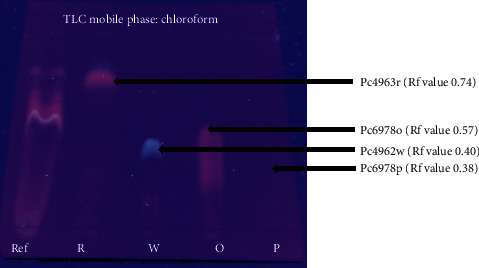
TLC plate showing the four isolated chromatograms as visualized under UV 254 nm. Spot labeled R is the chromatogram Pc4963r. W is the chromatogram Pc4962w. Spot O is the chromatogram Pc6978o. Spot labeled P is the chromatogram Pc6978p. Spot labeled Rf represents unpurified pools as a reference. Chloroform was the mobile phase.

**Figure 7:**
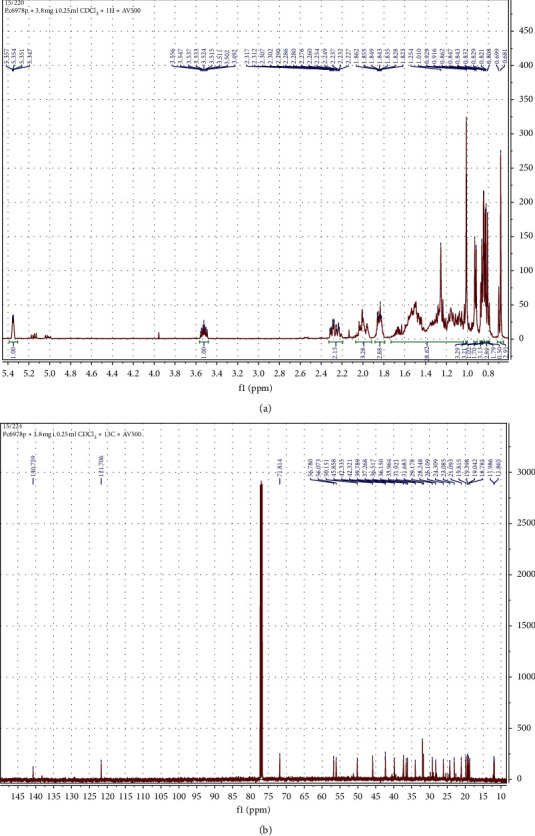
^1^H NMR (CDCl_3_, 500 MHz) spectrum of Pc6978p (a) and ^13^C NMR (CDCl_3_, 125 MHz) spectrum of Pc6978p (b).

The migrating chromatograms were isolated by scrapping off silica containing the targeted chromatograms from the glass plate. The composition of separated chromatograms on the scraped silica was eluted using chloroform through the Whatman No. 1 filter paper into different glass vials. The purity of each isolated chromatogram was determined using TLC. At least four compounds were obtained after elution of the four chromatograms from the silica. The four compounds showed different colors, red, white, orange, and purple, with Rf values of 0.74, 0.40, 0.57, and 0.38, respectively (Figure 6). The compounds were given code names Pc4963r, Pc4962w, Pc6978o, and Pc6978p (Figure 6). Only the contents of Pc6978p were shown to contain a single compound by showing a single spot on the TLC plate (Figure 6). The structure of the pure isolated Pc6978p was determined to be *β*-sitosterol (Figure 2) by NMR as confirmed by the ^1^H NMR (CDC13, 500 MHz) and ^13^C NMR (CDCl_3_, 125 MHz) spectrum of Pc6978p (Figure 7). The compounds showing more than one spot on the TLC were stored in glass vials for further analysis on a later stage.

### 3.4. In Vitro Bioactivities of the Purified Compounds

**Figure 8:**
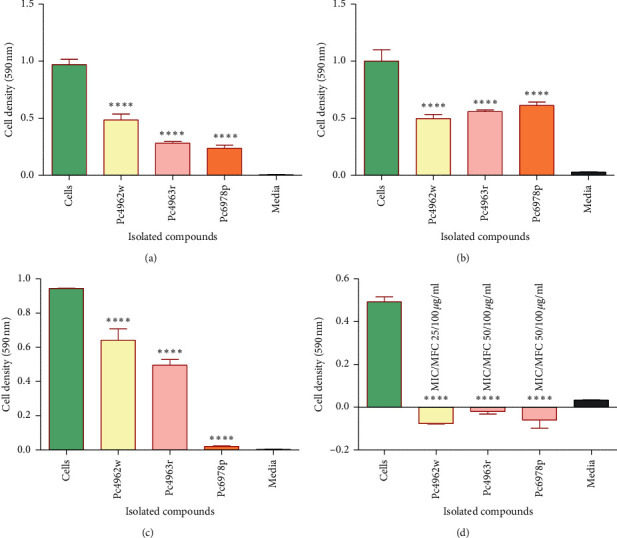
*In vitro* antibacterial effects of the isolated compounds at a concentration of 100 *µ*g/mL against *S. aureus* (a), *K. pneumoniae* (b), *M. smegmatis* (c), and *C. krusei* (d). “Media” column is the negative control showing no cell growth. “Cells” column is the positive control for cell growth. Error bars represent the standard deviation from mean (*n* = 4). The asterisk (∗) indicates the significant difference from the positive control using statistical one-way ANOVA (where *∗P* < 0.05, *∗∗P* < 0.01, *∗∗∗P* < 0.001, *∗∗∗∗P* < 0.0001).

Compounds Pc4963r, Pc4962w, and Pc6978p were selected for the evaluation of antimicrobial activities against the test organisms. Compound Pc6978o was left out due to very low yield, lower than 3 mg (Table 2).  Figure 8 shows a graph of the antimicrobial activities of Pc4963r, Pc4962w, and Pc6978p at concentration of 100 *µ*g/mL against test cells. The cell densities correspond to the growth of cells after incubation at 37°C overnight. Compound Pc6978p showed the highest reduction effects on the growth of *S. aureus* cells with a percentage antibacterial activity of 75.3%, followed by Pc4963r which had a percentage activity of 71.1% (Figure 8(a)). Compared to the other two isolated compounds, Pc4962w had the least antibacterial activity against *S. aureus*, with a percentage activity of 50.5% (Figure 8(a)). However, statistical analysis using the one-way ANOVA (where *P* < 0.05) has shown that all compounds had statistically significant reduction effects on growth of *S. aureus* (Figure 8(a)). Data analysis showed that all the compounds significantly reduced the growth of *K. pneumoniae* (Figure 8(b)). However, compared to the other two isolated compounds, Pc6978p had the least antibacterial activity against *K. pneumoniae*, with a percentage activity of 39% (Figure 8(b)). Compound Pc4962w showed the highest reduction effects on the growth of *K. pneumonia*e cells with a percentage antibacterial activity of 51%, followed by Pc4963r which had a percentage activity of 44% (Figure 8(b)). Compound Pc6978p showed the highest reduction effects on the growth of *M. smegmatis* cells with a percentage antibacterial activity of 97.9%, followed by Pc4963r which had a percentage activity of 46.8% (Figure 8(c)). Compared to the other two isolated compounds, Pc4962w had the least antibacterial activity against *M. smegmatis* with the percentage activity of 31.9% (Figure 8(c)). However, statistical analysis using the one-way ANOVA (where *P* < 0.05) has shown that all the compounds significantly reduced the growth of the *M. smegmatis* (Figure 8(c)). The antifungal activities of Pc4963r, Pc4962w, and Pc6978p at concentration of 100 *µ*g/mL against *C. krusei* cells were investigated. The graph in Figure 8(d) shows that all the three isolated compounds completely inhibit the growth of *C*. *krusei* cells.

### 3.5. Biocidal Activity Assays of *β*-Sitosterol (Pc6978p) on Mendy Fabric

**Figure 9:**
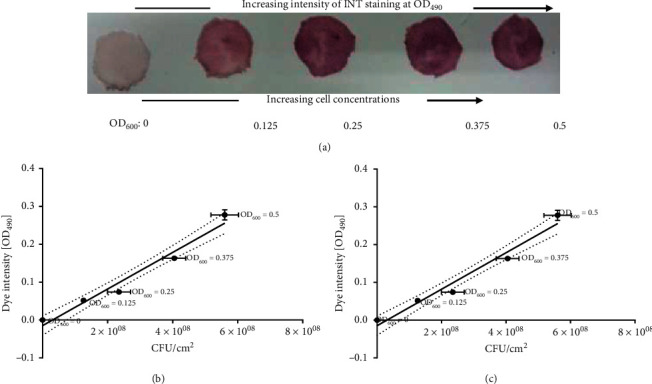
Standard curves of the INT staining intensity relative to the cell numbers. Solutions containing different numbers of *C. krusei* cells were applied onto Mendy fabric and stained with INT for 30 minutes (a). Bacteria/fungi CFU/cm^2^ (*x*-axis) are correlated to the intensity of INT staining at OD_490_ (*y*-axis). Initial OD_600_ of the applied fungal/bacterial solution is indicated for each data point. By applying a volume of 100 *µ*L, *C. krusei* with an initial OD_600_ of 0.5 was found to correspond to 5.6 × 10^8^ fungal cells per cm^2^ of fabric after 1 hour incubation (b), while an OD_600_ of 0.5 equaled 3.15 × 10^10^ *M. smegmatis cells* per cm^2^ (c). A linear regression (black line) through the background value precisely fits the staining intensity quantified for different cell numbers, indicating that there is no saturation of dye production in the tested cell densities. Error bars represent measurements for 4 individual fabric samples.

Pc6978p had the highest activity against *C. krusei* and *M. smegmatis.* Therefore, biocidal activity of compound Pc6978p was evaluated against *M. smegmatis* and *C. krusei*. Standard curves were first generated by adding different concentrations of cell on different pieces of fabric; the increase in color intensity corresponded to the increasing number of cells (Figure 9). A typical layout of the fabric used to generate the standard curves is shown in Figure 9(a). The color intensity of the dye on fabric was increasing from OD_490_ 0 to OD_490_ 0.5 indicating the increasing cell numbers attached to different pieces of Mendy fabric. Figures 9(b) and 9(c) show standard curves of the INT staining intensity relative to the *C. krusei* and *M. smegmatis* cell numbers, respectively.

**Figure 10:**
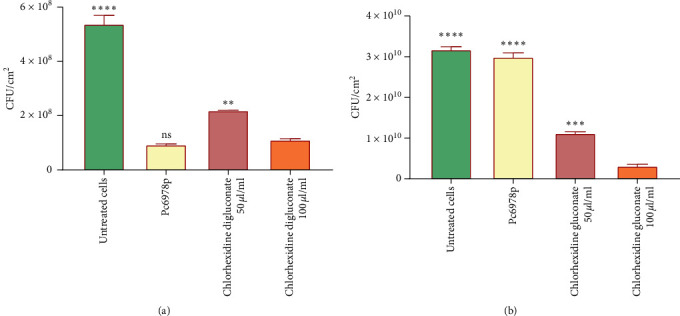
Biocidal effects of compound Pc6978p at a concentration of 100 *µ*g/mL on *C. krusei cells* (a) and *M. smegmatis* cells (b) after incubation for 30 minutes at 37°C. Untreated cells are the positive control of growth of cells; no biocide was added to the cell. Chlorhexidine digluconate 50 *µ*g/mL and 100 *µ*g/mL are the standard positive control of a biocide. Error bars represent the standard deviation of 2 individual replicates. The asterisk (*∗*) indicates the significant difference from the positive control chlorhexidine 100 *µ*g/mL using statistical one-way ANOVA (where *∗P* < 0.05, *∗∗P* < 0.01, *∗∗∗P* < 0.001, *∗∗∗∗P* < 0.0001, and “ns” means no significant deference).

At a concentration of 100 *µ*g/mL, compound Pc6978p had 83% biocidal activity against *C. krusei* attached to the Mendy fabric (Figure 10(a)). The compound removed and killed about 4.45 × 10^8^*C. krusei* cells per cm^2^ of Mendy fabric after 30 minutes of incubation. The positive control chlorhexidine digluconate 50 *µ*g/mL and 100 *µ*g/mL had 59% and 79% biocidal activity on *C. krusei* attached to the Mendy fabric (Figure 10). Statistical analysis using the one-way ANOVA showed that there was no difference between the biocidal activities of the positive control chlorhexidine digluconate at 100 *µ*g/mL and the compound Pc6978p. Pc6978p did not have significant biocidal activity, only producing 5.8% cell reduction against *M. smegmatis* attached to the Mendy fabric (Figure 10(b)).

However, the compound has been shown have biocidal activity of around 1.9 × 10^9^ *M. smegmatis* cells attached to 1 cm^2^ of Mendy textile after 30 minutes of incubation. The positive control chlorhexidine 50 *µ*g/mL and 100 *µ*g/mL had 64.7% and 90.1% biocidal activity on *K. pneumoniae* cells attached to the Mendy textile in only 30 minutes, which was significantly different from the activity of the compound Pc6978p (Figure 10(b)).

### 3.6. Structure Determination of the Isolated Compound

The structure of the compound Pc6978p was confirmed by the nuclear magnetic resonance (NMR) spectroscopy to be *β*-sitosterol (Figure 2) [31, 32]. The following are the spectroscopic characteristics of the compound Pc6978p: ^1^H-NMR (500 MHz, CDCl_3_) *δ*: 5.35 (1H, *brq*, *J* = 1.5, 2.0, 3.0 Hz, H-6), 3.52 (1H, *m*, H-3*α*), 1.01 (3H, *s*, Me-19), 0.92 (3H, *d*, *J* = 6.5 Hz, Me-21), 0.86 (3H, *t*, *J* = 5.0 Hz, Me-29), 0.84 (3H, *d*, *J* = 2.0 Hz, Me-26), 0.83 (3H, *d*, *J* = 4.0 Hz, Me-27), 0.68 (3H, *s*, Me-18) (Figure 7(a)).

^13^C-NMR (100 MHz, CDCl_3_) *δ*: 140.75 (*s*, C-5), 121.70 (*d*, C-6), 71.81 (*d*, C-3), 56.78 (*d*, C-14), 56.07 (*d*, C-17), 50.15 (*d*, C-9), 45.85 (*d*, C-24), 42.33 (*s*, C-13), 42.32 (*t*, C-4), 39.78 (*t*, C-12), 37.26 (*t*, C-1), 36.51 (*s*, C-10), 36.15 (*d*, C-20), 33.96 (*t*, C-22), 31.92 (*t*, C-7), 31.88 (*d*, C-8), 31.68 (*t*, C-2), 29.17 (*d*, C-25), 28.24 (*t*, C-16), 26.10 (*t*, C-23), 24.30 (*t*, C-15), 23.08 (*t*, C-28), 21.09 (*t*, C-11), 19.81 (*q*, C-26), 19.39 (*q*, C-19), 19.04 (*q*, C-27), 18.78 (*q*, C-21), 11.98 (*q*, C-18), 11.86 (*q*, C-29) (Figure 7(b)). HRESI-MS: *m/z* 414.4031 (calcd. for C_29_H_50_O, 414.3861). mp = 137.8–138.7°C.

## 4. Discussion

In this study, *β*-sitosterol was isolated from *P. curatellifolia* extracts during extraction to determine active antimicrobial and biocidal compounds from this plant. Both fungi and bacteria remain major life threatening pathogens in humans, mostly to the immunocompromised or immunodeficient patients [33]. Despite the existence of potent antibiotic and antifungal agents, resistant and multiresistant strains are continuously emerging, imposing the need for a permanent search for alternative microbial agents [34]. Moreover, disinfection proved to be a frontline strategy of controlling microbial contamination and spread and development of resistance [35]. Therefore, this study was made to assess both the microbial and biocidal activity of phytochemicals from *P. curatellifolia*, as plants extracts are considered to be one of the best sources of alternative antimicrobials [36].

However, it is known that the activity of the plant extracts could be due to the different classes of phytochemicals present, their quantities, and the possible interactions with other constituents of the extract [1]. Furthermore, antimicrobial properties of plant extracts were observed to be influenced by several factors including the sample preparation and the extraction process [16, 36]. Therefore, in the study, precautions were taken to ensure that potential active phytochemicals were not lost, distorted, or destroyed during the extraction. These include air-drying the sample at room temperature rather than oven drying at 60°C [16]. The effects of heat on potential active compounds were demonstrated by the significant reduction in the active anthocyanin content in some leaf extracts, possibly due to accelerated chalcone formation on exposure to high temperatures [37]. It was reported that bioactive phytochemicals, such as sinensetin and rosmarinic acid content, were affected by the oven and sunlight drying [38].

Maceration extraction technique based on the extracting power of different solvents was utilized in this study [16]. This technique targeted extraction of a wide range of compounds from the leaf material; hence, a combination of the total extraction (DCM-MeOH) and the serial exhaustive method was used on the same sample of *P. curatellifolia.* The DCM-MeOH extraction had the highest percentage yield, 5.53%. This extraction technique uses a cosolvent extraction system of dichloromethane (nonpolar) and methanol (polar). The method was designed to extract both the polar and nonpolar compounds in a single extraction process, which justify the high yield compared to the polarity specific extraction methods [39].

In the polarity specific serial exhaustive methods used in this study, the methanol extraction had the highest yield, 3.31%, followed by the water extract that had 3.0%. The general observation was that the extraction yield increased as the polarity of the solvents increased. Increase in yield may be an indication of the presence of larger amounts of polar compounds in *P. curatellifolia* leaves compared to nonpolar compounds. Higher amounts of polar compounds are in accordance with previous observations that the dominant compounds in plants leaves are polar in nature [40].

Bhunu et al. [28] used a similar extraction method to extract phytochemicals from *P. curatellifolia.* Similar to the result shown in this study, their study [28] has also shown that the DCM-MeOH extraction had a higher yield compared to the serial exhaustive extraction. Reduction in yield of serial exhaustive extraction in this study may be probably due to the fact that the serial exhaustive extraction was done on the same plant materials that have some or most of the extracts already extracted by the DCM-MeOH extraction.

Furthermore, it was noted that the yield of DCM-MeOH extraction and the combined yield of the serial exhaustive extraction in the study by Bhunu et al. [28] were higher than the yields produced from the same extraction in the current study. Their study [28] has shown that the DCM-MeOH extraction yield was as high as 18% compared to 3.5% percent of this recent work. They also produced 12% of the combined serial exhaustive extraction compared to only 9% from the current study. Reduced yield in the current study is probably due to the material to solvent ratio of the extract. Bhunu et al. [28] used 1:10 (*w/v*) ratio for extraction and produced better extraction yield compared to 1:3 (*w/v*) ratio used in this study.

These observations have justified the importance of careful selection of the extraction methods and solvent ratios for better phytochemicals extraction [41]. Since these two studies have used almost the same extraction methods and solvents produced different yields. The observation was that extraction efficiency in maceration of extraction is also affected by the solubility of the solid plant material and effective diffusion in the liquid extraction solvent [42]. The higher the solid to solvent ratio, the higher the yield [42]. Moreover, all stages of extractions, from the pre-extraction and extraction, are equally important in studying plant phytochemicals [41]. Sample preparation, such as grinding and drying, was also shown to affect the efficiency and phytochemical constituents of the final extractions, which eventually have an effect on the final extracts [16].

*In vitro* bioactivities of all the seven crude extracts were evaluated against the Gram-positive *S. aureus*, the Gram-negative *K. pneumoniae*, the acid-fast *M. smegmatis*, and the fungus *C. krusei* before fractionation and isolation of single compounds. The choice of purification of compound used in this study was activity-guided. According to Kuete et al. [43], antibacterial activity of a crude plant extract has been defined as significant when MIC is below 100 *µ*g/mL, moderate when 100 *µ*g/mL < MIC > 625 *µ*g/mL, or low when MIC > 625 *µ*g/mL. The antimicrobial activity of a compounds is defined as significant when MIC is below 10 *µ*g/mL, moderate when 10 *µ*g/mL < MIC > 100 *µ*g/mL, or low when MIC >100 *µ*g/mL [43]. Therefore, the bioactivity assay in this study was guided by those standards. Extracts were considered to be significantly active when MIC values below 100 *µ*g/mL were obtained on at least one tested organism. Interestingly, 5 out of 7 extracts were active against at least one test microbe.

*M. smegmatis* was the most susceptible to most of the *P. curatellifolia* leaf extracts, with the ethanol and methanol extract being the most active extracts against *M. smegmatis* cell growth, with an MIC of 25 *µ*g/mL. The DCM-MeOH extract and the acetone extract had an MIC of 50 *µ*g/mL on *M. smegmatis*. The activities of extracts against *M. smegmatis* were observed to generally increase as extraction solvent polarity increased from hexane to methanol. Out of the four extracts that inhibited the growth of *M. smegmatis* cells, only the DCM-MeOH and the methanol extract had MBC values of less than or equal to 100 *µ*g/mL against *M. smegmatis*. This observation indicates possible bactericidal effects of both the DCM-MeOH and the methanol extract again *M. smegmatis* at concentration of 100 *µ*g/mL, since the two extracts completely inhibited the visible *M. smegmatis* growth on the agar plates after incubation at 37°C for 24. The acetone and ethanol extracts were bacteriostatic against *M. smegmatis* at a concentration less than or equal to 100 *µ*g/mL. Significant bactericidal effects of DCM-MeOH extracts against *M. smegmatis* may be because of synergism of different compounds since the DCM-MeOH extracts contained a wide range of compounds including both polar and nonpolar compounds.

Bhunu et al. [28] also demonstrated susceptibility of *M. smegmatis* cells to *P. curatellifolia* leaf extracts. According to their study [28], the MIC of the *P. curatellifolia* leaf extracts was 6.2 *µ*g/mL for the acetone extract, 12.5 *µ*g/mL for both the ethanol and the DCM-MeOH extracts, and 50 *µ*g/mL for both the methanol and ethyl acetate extracts. The MIC values from the study by Bhunu et al. [28] were slightly lower than the value obtained in this study. This may be due to factors including the effectiveness of the extraction and preparative methods [16]. Susceptibility of *M. smegmatis* to *P. curatellifolia* leaf extracts may be due to the destruction of waxy cell envelop on mycobacteria [42]. The waxy cell envelop is known to be innate, making the mycobacteria the least susceptible to antimicrobial agents [42]. However, some phenolics, e.g., the gallic acid, were demonstrated to interact with these membranes envelops [42].

The hexane extract was among the extracts that showed significant antimicrobial activity according to [43] standards. Despite being less potent to all the bacterial test organisms, the hexane extract showed significant antifungal activity against *C. krusei*. This may be an indication that nonpolar extracts of *P. curatellifolia* have less antibacterial activities but stronger antifungal. The MIC of the hexane extract on *C. krusei* was 25 *µ*g/mL. The extract was not fungicidal at a concentration less than or equal to 100 *µ*g/mL. Interestingly, of all the extracts, only the hexane extract showed potent antifungal activity. Silva Sa et al. [44] have shown that nonpolar fractions including the hexane and dichloromethane fraction from *Myrcia tomentosa* (Aubl.) DCM leaf extracts showed antifungal activity against *Candida* species with low MIC values, which ranged from 4 to 256 *µ*g/mL. Therefore, it is most likely that the constituents of the nonpolar extracts are responsible for the anti-*Candida* activity of *P. curatellifolia.* The probable reason for the strong activity of hexane extract against the *C. krusei* may be the interaction between *C. krusei* cells and nonpolar compounds, since the strong hydrophobicity in the *C. krusei* cell membrane allows stronger affinity to nonpolar compounds [45]. In a similar study, Samie et al. [46] reported that hexane extracts were among the active extracts against *C. krusei*. Samie et al. [46] went on to conclude that most *Candida* species were susceptible to plant hexane extracts suggesting that many of the antifungal components of these plants were nonpolar compounds [45]. Lack of significant antibacterial activity by the hexane extract with some solvents showing activity is in accordance with the observations by Peni et al. [22], who showed that different solvent extracts of the same plant may have different pharmacological properties.

None of the extracts used in this study were able to completely inhibit both the Gram-positive *S. aureus* and Gram-negative *K. pneumoniae* at concentration less than or equal to 100 *µ*g/mL. Lack of activity below 100 *µ*g/mL of extracts against *S. aureus* was previously shown by Oshomoh and Idu [47], who indicated that the least MIC of some *P. curatellifolia* extracts was 3.125 mg/mL against *S. aureus*. However, the results from this study have shown that the DCM-MeOH extract had considerable activity against Gram-positive cells at a concentration of 100 *µ*g/mL. At that concentration, the DCM-MeOH extract inhibited up to 90% of the growth of *S. aureus* cells. The activities of most extracts against *S. aureus* were generally above 50%. However, the higher activity against *S. aureus* in this study was generally observed with polar *P. curatellifolia* extracts. These observations were in agreement with those by Peni et al. [22].

The DCM-MeOH had the highest activity against the Gram-negative *K. pneumoniae* inhibiting up to 74.8% of cell growth. A previous study by Halilu et al. [48] showed that the MIC of *P. curatellifolia* extracts against some Gram-negative microbes was above 100 *µ*g/mL limit. The least MIC of *P. curatellifolia* extracts against *P. aeruginosa* was 1.5 mg/mL [48]. According to Halilu et al. [48] *P. curatellifolia* extracts were more active against Gram-positive compared to the Gram-negative microbes. The results from the study by Halilu et al. [48] agree with those from this study, showing that the DCM-MeOH leaf extracts from *P. curatellifolia* had a higher activity against *S. aureus* (89.9%) compared to *K. pneumoniae* (74.5%). The susceptibility of the Gram-positive bacteria may be due to the disruption of the cell walls by some phytochemicals present in the crude extracts commonly classified as phytoanticipins [49].

After evaluating the activity of the crude extracts, extracts that showed considerable activity against at least one of the test microbes were selected for fractionation and isolation of pure compounds. The hexane, methanol, ethanol, and DCM-MeOH were selected for fractionation. A chromatogram with more than a single spot on the TLC was considered not pure. The purple compound Pc6978p (Rf 0.38) was considered pure since only a single spot was observed on the TLC plate. After elution of the chromatograms from the preparative TLC silica, compound Pc6978p was concentrated to a white crystalline powder. Pc4963r, Pc4962w, and Pc6978o were concentrated to green crystalline powder, white crystalline powder, and green crystalline powder, respectively. The structure of Pc6978p was determined to be *β*-sitosterol on the basis of ^1^H and ^13^C NMR spectroscopic data and by comparing them to those reported in the literature [50].

*β*-Sitosterol is one of most spread phytosterols in the plant kingdom [23]; *β-sitosterol* was isolated previously from a number of plant species [51–53], but to the best of our knowledge, *β-sitosterol* was isolated for the first time from *P. curatellifolia* extracts. *In vitro* bioactivities of *β-sitosterol* were evaluated against the four test organisms. The isolated *β-sitosterol* failed to show significant activity against all the test organisms according to Kuete [43] proposed standards. Nonetheless, the compound managed to reduce the growth of *M. smegmatis* cells by 97.9%. There is a notable loss of activities of Pc6978p compared to the activity of some crude extracts (DCM:methanol, acetone, ethanol, and methanol) against *M. smegmatis*. This may be attributed to loss of synergism of compounds in the crude extracts through purification. Synergistic effects occur when two compounds increase one another's potency, resulting in mixtures that have stronger effect than predicted based on activities of their components in isolation [54]. High antimicrobial activity of some crude extracts observed may be due to the synergistic effects of the compounds present in the extracts [54]. For example, combinations of thymol and eugenol were shown to have synergistic effects against *E. coli* [55].

*β*-Sitosterol reduced the cell growth of the Gram-positive *S.aureus* and Gram-negative *K. pneumoniae* by 75% and 39%, respectively, at 100 *µ*g/mL. It was also noted that the isolated *β-sitosterol* has significantly reduced activity against these two microbe as compared to the activity of the DCM:methanol and methanol extracts. This may be an indication that the most active compounds against Gram-positive and Gram-negative bacteria may be more in polar extracts (e.g., methanol extracts) than in nonpolar ones, since some studies have reported the isolation of *β*-sitosterol in nonpolar solvents [23]. Loss of activity of the isolated *β*-sitosterol may also be due to loss of synergistic effects of compounds present in extracts against *S. aureus* and *K. pneumoniae.*

While the activity of *β*-sitosterol cannot be classified as significant [43], results from this study showed significant activity of this compound against all the test bacterial strains. The observed antibacterial activity of the isolated compound are in agreement with the reported antibacterial activity of *β*-sitosterol against the same bacterial strains [52]. Using the disc-diffusion method [52], other researchers have also reported low activity of *β-*sitosterol against both the Gram-negative and Gram-positive cells. The growth inhibition in that study was 11 mm and 13. 5 mm against *S. aureus* and Gram-negative *E. coli*, respectively, using the disc-diffusion method [52]. There are also reports that show antibacterial activity of *β*-sitosterol against several bacterial species [23, 56]. Therefore, from this study, it was observed that *β-*sitosterol has activity against the test bacterial cells, although the activity can only be classified as low according to Kuete [43]. Low but statistically significant activity of the *β*-sitosterol against Gram-positive, Gram-negative, and acid-fast bacteria has highlighted a degree of the broad-spectrum activity of the *β*-sitosterol. However, of all the activities of *β-*sitosterol, the lowest observed was against Gram-negative bacteria.

*β*-Sitosterol had moderate activity against *C. krusei* with an MIC value of 25 *µ*g/ml and MFC of 50 *µ*g/mL against *C. krusei.* The results confirm reports suggesting that phytosterols do have an antifungal potential [57]. From this study, an increase in in activity against *C. krusei* was observed between the activity of the most active hexane extract and activity of the purified compound. The hexane extract failed to completely inhibit the growth of *C. krusei* after incubation at a concentration of 100 *µ*g/mL for 18 hrs, while the pure compound was able to kill all the cells at 50 *µ*g/mL in the same incubation conditions.

The enhancement of antimicrobial activity of *β*-sitosterol as a single entity chemical may be due to the elimination of the antagonistic effects of different compounds found in crude extracts. Antagonistic effects occur when two compounds inhibit one another's activities, such that mixtures are less effective than predicted based on the activities of each compound in isolation [55]. Antagonistic effects of different compounds in extracts are known to reduce the activity of some active compounds [54]. For example, eugenol and thymol had antagonistic effects against *Crithidia fasciculata* [58]. Hence, purification of some compounds is sometimes recommended to achieve the highest antimicrobial activity of some phytochemicals [58].

The antifungal activity of *β*-sitosterol observed in this study is in agreement with a previous study reporting that *β*-sitosterol inhibited the growth of *C. albicans* and *C. krusei* at 50 *µ*g/mL [59]. Although some studies have indicated that *β*-sitosterol was fungistatic rather than fungicidal [24], this study has shown that *β*-sitosterol was fungicidal at 50 *µ*g/mL. However, from these observations, further analysis of *β*-sitosterol on the anti-*Candida* species, especially *C. krusei*, may be necessary since this fungal species is clinically significant. *C. krusei* induces candidiasis which is also caused by many other species of the *Candida* genus [60]. *C. krusei* is also listed as emerging fungal nosocomial pathogen whose infection is common among immunocompromised patients and those with hematological malignancies [60].

Therefore, further analysis may be useful to determine if the *β*-sitosterol can be used to treat such fungal related ailment, as well as to unlock more of its value in human health and hygiene. For example, its synergistic potential can be used to enhance the activity of already present antimicrobial agents to minimize microbial resistance. The isolated *β*-sitosterol can also be used as an additive to present antifungal agents. Additive effects indicate that the effects of chemicals are independent of one another [55] but have similar modes of action, such that adding a second compound has the same effect as adding more of the first compound [61]. A clinical example of additive effects due to independent actions of phytochemicals, artemisinin, and curcumin against malaria was descried by Nandakumar et al. [62].

Chemical composition and structures of the contents in Pc4963r, Pc4962w, and Pc6978o could not be determined in this study. However, antimicrobial activities of Pc4963r and Pc4962w were evaluated against all the test organisms. Antimicrobial activities of Pc6978o were not evaluated due to very low yield. The highest activities of both Pc4963r and Pc4962w were observed against *C. krusei* cells; both compounds showed moderate activity against *C. krusei*. Pc4962w had MIC and MFC values of 25 *µ*g/mL and 100 *µ*g/mL, respectively. Pc4963r had MIC and MFC values of 50 *µ*g/mL and 100 *µ*g/mL against *C. krusei*. The study has shown an increase in activity against *C. krusei* between the activity of the most active hexane extract and the activity of both Pc4963r and Pc4962w. The hexane extract failed to totally eliminate the *C. krusei* cells following incubation at a concentration of 100 *µ*g/mL for 18 hours. On the other hand, Pc4963r and Pc4962w were fungicidal cells at 100 *µ*g/mL under the same incubation conditions. Improvement in activity of both compounds may be due the elimination of the antagonistic effects of different compounds found in crude extracts. The similarity of these results to those of *β*-sitosterol coupled with the fact that *β*-sitosterol, Pc4963r, and Pc4962w were isolated from closely related pools may lead to the suggestion that both Pc4963r and Pc4962w may also be phytosterols. This suggestion has been derived from a previous suggestion that most phytosterols have an antifungal potential [49].

After determination of the biological activity of the isolated compound, the study went on to determine the applicability of the obtained results. Therefore, following the activity-guided approach of analysis, biocidal effects of Pc6978p were evaluated against *C. krusei* cells and *M. smegmatis* on the Mendy fabric. The Mendy fabric is the most used material to make uniforms for nurses in most Zimbabwean hospitals. However, it was noted that, in most hospital setups, nurses are the most vulnerable to microbial contamination since they are the primary handlers of infected and sick people [63]. In addition, nurses spend most of the time in sick people's wards, which may increase the chances of cloth contamination by infection pathogens [63].

Microbial contamination of health workers' cloths, surfaces, and equipment plays a significant role in the spread of pathogens [15]. Successful transmission of pathogens depends on many factors, including the capacity of microorganisms to remain viable on dry surfaces, their resistance to disinfectants, and the frequency at which infected surfaces or equipment comes in contact with patients and healthcare staff [15]. Furthermore, antimicrobial resistance could be acquired through exposure of microbes to deferent conditions and substances while being attached to textiles and surfaces [64, 65]. Therefore, evaluation of possible alternative sources of biocidal compounds was investigated in this study.

Compound Pc6978p had 86% biocidal activity on *C. krusei* cells attached to the Mendy fabric. That is, at a concentration of 100 *µ*g/mL, *β*-sitosterol removed and was fungicidal to about 5.04 × 10^8^ *C. krusei* cells per cm^2^ of Mendy fabric after 30 minutes of incubation. These observations show that Pc6978p had better biocidal activity compared to the known biocide chlorhexidine digluconate which had 80% activity against the same cells under the same condition. However, there was no statistically significant difference between the biocidal activity of Pc6978p and chlorhexidine digluconate. From this study, it can be proposed that the compound can be a candidate for the formulation of alternative biocides used to decontaminate textiles. A study by Diba [66] has shown that hospital textiles, mostly blankets, are the main sources of *C. krusei* contamination. Several studies indicate that different *Candida* species can have similar adherence properties to cotton and other fabrics [66], as well as to plastics and other synthetic materials [66]. Furthermore, it is suggested that significant environmental contamination in hospital setup is from *Candida* species [66], especially antifungal *C. krusei* [67]. It is possible that the strong hydrophobicity in the *C. krusei* cell membrane allows stronger attachment to nonpolar surfaces and these include catheter surfaces, compromising sterility and differential diagnosis [35]. Therefore, *β*-sitosterol can also be formulated into some of the disinfectants that are used in hospital settings to reduce *C. krusei* contamination. Hence, further studies on the biocidal effects of Pc6978p against *C. krusei* on other surfaces are recommended.

Even though compound Pc6978p showed high growth reduction effects on *M. smegmatis* in *in vitro* antimicrobial assays, it failed to exhibit biocidal activity against the *M. smegmatis* attached to the Mendy textile. Pc6978p only reduced 6% of the viable cells compared to the positive control chlorhexidine digluconate that had 90% biocidal activity under the same conditions. The observations may suggest that Pc6978p is less effective as a biocide against *M. smegmatis*. However, it is important to note that the effectiveness of biocides is influenced by many factors, such as concentration, contact time, and environmental conditions to which it is applied [68, 69]. The condition, whether in suspension, adhering to a surface, or in a biofilm, would have an effect on the effectiveness of biocides [70]. Therefore, all these effects should be investigated in future, so that full biocidal potential of *β*-sitosterol and other natural compounds from this plant can be exploited.

## 5. Conclusion

*P. curatellifolia* leaf extracts, extracted in this study, have been shown to contain phytochemicals that inhibit the growth of test bacterial and fungal species. This is in agreement with the use of this plant in traditional medicine. The DCM-methanol and ethanol extracts were the most active against most test bacterial species. *Mycobacterium smegmatis* was the most susceptible to most extracts. *β*-sitosterol showed fungicidal activity against *C. krusei* and potent biocidal activity against *C. krusei* cells attached to the Mendy textile. However, further work has to be done to determine if the isolated compounds can be used to treat fungal and bacterial related ailment in model system, as well as to unlock their potential use in human health industry as biocides.

## Figures and Tables

**Table 1:** Extraction yield of the *P. curatellifolia* leaves.

Extraction solvent	Extract mass (g)	Percentage yield (%)
Dichloromethane:methanol	53.0	3.53
Hexane	3.25	0.23
Ethyl acetate	5.70	0.41
Ethanol	32.5	2.46
Methanol	43.0	3.31
Water	30.0	3.0

**Table 2:** Antimicrobial activities of the extracts on the test organisms expressed as % inhibition at 100 (*µ*g/mL)/MIC (*µ*g/mL)/MBC (*µ*g/mL).

Extracts	*S. aureus*	*K. pneumoniae*	*M. smegmatis*	*C. krusei*
DCM:methanol	89.9/-/-	74.8/-/-	100/50/50	0/-/-
Hexane	54.9/-/-	37.5/-/-	55.3/-/-	100/25/50
Ethyl acetate	3.6/-/-	54.2/-/-	70.3/-/-	16.9/-/-
Acetone	50.1/-/-	47.9/-/-	100/50/-	0/-/-
Ethanol	51.8/-/-	50.7/-/-	100/25/-	33.6/-/-
Methanol	81.5/-/-	52.5/-/-	100/25/100	21.9/-/-
Water	50.2/-/-	26.9/-/-	75.3/-/-	64/-/-

MIC: minimum inhibitory contraction. “-” means that MIC/MBC/MFC was greater than 100 *µ*g/mL. “0” means no inhibition.

## Data Availability

The datasets used and/or analyzed during the current study are available from the corresponding author on reasonable request.
